# Circulating MicroRNAs in Plasma of Hepatitis B e Antigen Positive Children Reveal Liver-Specific Target Genes

**DOI:** 10.1155/2014/791045

**Published:** 2014-12-17

**Authors:** Thilde Nordmann Winther, Kari Stougaard Jacobsen, Aashiq Hussain Mirza, Ida Louise Heiberg, Claus Heiner Bang-Berthelsen, Flemming Pociot, Birthe Hogh

**Affiliations:** ^1^Department of Paediatrics, Hvidovre Hospital, University of Copenhagen, Kettegaard Allé 30, 2650 Hvidovre, Denmark; ^2^Department of Paediatrics and Center for Non-Coding RNA in Technology and Health, Herlev Hospital, University of Copenhagen, Arkaden, 2730 Herlev, Denmark

## Abstract

*Background and Aim*. Hepatitis B e antigen positive (HBeAg-positive) children are at high risk of severe complications such as hepatocellular carcinoma and cirrhosis. Liver damage is caused by the host immune response to infected hepatocytes, and we hypothesise that specific microRNAs play a role in this complex interaction between virus and host. The study aimed to identify microRNAs with aberrant plasma expressions in HBeAg-positive children and with liver-specific target genes. *Methods*. By revisiting our previous screen of microRNA plasma levels in HBeAg-positive and HBeAg-negative children with chronic hepatitis B (CHB) and in healthy controls, candidate microRNAs with aberrant plasma expressions in HBeAg-positive children were identified. MicroRNAs targeting liver-specific genes were selected based on bioinformatics analysis and validated by qRT-PCR using plasma samples from 34 HBeAg-positive, 26 HBeAg-negative, and 60 healthy control children. *Results*. Thirteen microRNAs showed aberrant plasma expressions in HBeAg-positive children and targeted liver-specific genes. In particular, three microRNAs were upregulated and one was downregulated in HBeAg-positive children compared to HBeAg-negative and healthy control children, which showed equal levels. *Conclusion*. The identified microRNAs might impact the progression of CHB in children. Functional studies are warranted, however, to elucidate the microRNAs' role in the immunopathogenesis of childhood CHB.

## 1. Introduction

Children with chronic hepatitis B (CHB) have a lifetime risk of developing hepatocellular carcinoma (HCC) up to 25% and an incidence of cirrhosis of 2-3% per year [1, 2]. It is widely accepted that the natural course of CHB is determined by the host-virus interaction; however, the exact mechanisms responsible for disease progression in children are not fully understood.

Evidence suggests that microRNAs play a role in the complex interaction between the hepatitis B virus and host [3]. Our group recently identified a panel of 16 microRNAs aberrantly expressed in plasma of children with CHB and suggested a potential role of these microRNAs in the pathogenesis of childhood CHB [4].

Risk of progressive liver disease primarily applies to hepatitis B e antigen positive (HBeAg-positive) children and seroclearance of HBeAg is a key event in the natural course of disease [5]. Most children who undergo HBeAg seroconversion are defined inactive carriers, with absent or low viral replication, and usually inactive liver histology [5]. Inactive carriers with no signs of cirrhosis at seroconversion do not show disease progression over long-term follow-up (24–29 years) [6–8]. In the present study we hypothesise that microRNAs aberrantly expressed in plasma of HBeAg-positive children might be involved in the development of progressive liver disease.

It is well known that specific host factors in the liver tissue are tightly regulated in patients with CHB [9]. MicroRNAs may regulate specific individual targets or function as master regulators of cellular processes, and many microRNAs regulate their targets cooperatively [10, 11]. Identifying liver-specific target genes for aberrantly expressed microRNAs in HBeAg-positive children may provide better insight in understanding the role of microRNAs in the pathogenesis of childhood CHB.

The present study aimed to identify circulating microRNAs targeting liver-specific genes—specifically in HBeAg-positive children. Our data provide an important resource for future investigations aiming at deciphering the role of specific microRNAs in the pathogenesis of childhood CHB.

## 2. Patients and Methods

### 2.1. Patients and Healthy Controls

CHB is a notifiable disease in Denmark [12] and the definition of CHB is hepatitis B surface antigen (HBsAg) seropositivity for more than six months [13]. Danish children with CHB are mainly adoptees and immigrants from highly endemic countries. During the period from July 2005 to April 2011, all children aged 0–18 years with CHB who were reported to Statens Serum Institut, Copenhagen, Denmark (*n* = 202), were invited to participate in the study. Parents of 60 children volunteered, and these children were included in the study. The cohort of children with CHB was previously described [4].

Characteristics of the chronically HBV infected children in brief included mean age: 10.1 years (SD ± 3.9 years, range 0.9–17.3 years); gender: 26 males and 34 females; racial origin: 62% (*n* = 37) were Asian, 17% (*n* = 10) were African, and 22% (*n* = 13) were Caucasian.

Of the 60 children studied, 34 were HBeAg-positive and 26 were HBeAg-negative (and anti-HBe positive). Mean alanine aminotransferase (ALT) and HBV DNA (± SD) were as follows: HBeAg-positive: ALT 47 ± 28 U/L and HBV DNA 5*E* + 08 ± 1*E* + 09 IU/mL, and HBeAg-negative: ALT 26 ± 13 U/L and HBV DNA 9*E* + 02 ± 2*E* + 03 IU/mL. Among HBeAg-positive children, genotypes A, B, C, D, E, and F were identified, and among HBeAg-negative children, genotypes A, B, C, D, and E were identified. Eleven out of 26 HBeAg-negative patients were not successfully genotyped due to low HBV DNA levels (Table 1) [4]. None of the children showed symptoms of their HBV infection, and none of them had received antiviral treatment. The children were followed in accordance with international guidelines [13–15]. All patients were negative for HIV, hepatitis A virus, and hepatitis C virus.

Furthermore, 60 healthy control children (previously described [4]) were included between August 2010 and August 2011. These children were recruited prior to elective surgery for a hernia or prior to minor orthopaedic surgery at Hvidovre Hospital, University of Copenhagen, Denmark.

Characteristics of the healthy controls in brief are mean age: 7.1 years (SD ± 3.7, range 0.7–15.7); gender: 33 males and 27 females; racial origin: 15% (*n* = 9) were Asian, 2% (*n* = 1) were African, and 83% (*n* = 50) were Caucasian (Table 1) [4]. The children were tested HBsAg-negative and none of the children had received vaccination against HBV. The children were free from known medical conditions. Blood samples were obtained prior to anaesthesia.

### 2.2. Ethical Considerations

The study was performed in accordance with the criteria of the Helsinki II Declaration and was approved by the Ethics Committee, Capital Region of Denmark, Reference number H-KF-255584, and the Danish Data Protection Agency, Journal number 2009-41-4193. Parents of all participants provided informed written consent prior to any study procedure.

### 2.3. Blood Samples

Blood samples were processed as previously described [4]. Briefly, blood samples were collected in EDTA tubes, centrifuged at 2,500 g for 10 minutes at room temperature, separated, aliquoted, and stored at −80°C until further use.

### 2.4. HBsAg Quantification

HBsAg was quantified as previously described [4], using ARCHITECT HBsAg assay (Abbott, Chicago, IL, USA) according to manufacturer's instructions.

### 2.5. Screen of Plasma MicroRNA Levels in Children with CHB and in Healthy Controls

Recently, our group published an initial screen of plasma microRNA levels in HBeAg-positive, HBeAg-negative, and healthy children [4]. Briefly, microRNA polymerase-chain-reaction (PCR) panels (human panel I and II V2.M/R), miRCURY LNA Universal RT PCR system (Exiqon, Vedbaek, Denmark) were employed to measure plasma levels of 739 human microRNAs in a total of three samples: one sample contained plasma from 10 HBeAg-positive children, one sample contained plasma from 10 HBeAg-negative children, and one sample contained plasma from 10 healthy controls. Analyses were performed per Exiqon's instructions. According to the manufacturer, the microRNAs covered in the microRNA PCR panels are generally higher expressed and more likely differentially expressed in disease, or more often cited in the literature.

### 2.6. Selection of Candidate MicroRNAs

For selection of candidate microRNAs we followed stringent criteria and included only data from HBeAg-positive and HBeAg-negative children. Firstly, based on raw data, microRNAs with *C*_*T*_ values above 35 in HBeAg-positive and/or HBeAg-negative children were excluded. Secondly, raw data were normalised using three differentapproaches: global mean, U6, and geometric mean of miR-22-5p, -26a-5p, and 221-3p as previously described [4]. By using the comparative *C*_*T*_ method, the relative expression of each microRNA between the groups was calculated [16]. We focused on microRNAs aberrantly expressed in HBeAg-positive children and identified the top up- and downregulated microRNAs for further analyses.

### 2.7. Bioinformatics Analysis

Bioinformatics analysis was performed on all candidate microRNAs identified by revisiting our previous screen. Our analysis included retrieval of microRNA target genes with CLIP-Seq (cross-linking immunoprecipitation-high-throughput sequencing) overlap from starBase (sRNA target Base, release 2.1) and subsequently liver-specificity filters were applied on the retrieved microRNA target genes with CLIP-Seq overlap.

### 2.8. Retrieval of MicroRNA Target Genes with CLIP-Seq Overlap

MicroRNA target genes were retrieved using the starBase, a database that allows a comprehensive exploration of microRNA-target gene interaction maps from CLIP-Seq and Degradome-Seq data [17]. The predicted microRNA-target gene interactions in starBase are processed from five target prediction tools (TargetScan, PicTar, PITA, miRanda, and RNA22) overlapping with the CLIP-Seq data. Only those target genes that intersected the CLIP-Seq data sets with a biological complexity ≥2 (a measure of reproducibility between biological replicates or experiments to further reduce false positives) were retrieved.

### 2.9. Liver-Specificity Filter for MicroRNA Target Genes

Human liver-specific genes were retrieved from the TiGER (Tissue Specific-Gene-Expression and Regulation, version 1.0) [18] and TiSGeD (Tissue-Specific Genes Database) [19] databases. The TiGER database encompasses human tissue-specific gene expression profiles or expressed sequence tag (EST) data, cis-regulatory module (CRM) data, and combinatorial gene regulation data for interacting transcription factor (TF) pairs.

TiGER data is based on analyses of 30 human tissues identifying tissue-specific genes (specificity is determined by expression enrichment scores and associated −log10 (*P* value)), TFs and CRMs. A gene is defined as tissue-specific if it satisfies the following two conditions: enrichment score >5 and *P* value < 10^−3.5^. In case of the TiSGeD, tissue-specific genes are based on biomedical literature and data mining of gene expression profiles for over 100 human tissues. In TiSGeD, relative tissue-specificity of a gene is based on a statistical parameter, SPM, to quantitatively measure the specificity of a gene over tissues. An SPM parameter is a sensitive indicator in quantitative estimation of gene expression patterns. SPM ranges from 0 to 1.0. A value close to 1.0 indicates high tissue specificity of a gene.

From the TiGER database, liver-specific genes were retrieved: 383 genes based on ESTs (309 nonredundant genes); 300 CRM detections (105 nonredundant genes); and 160 TF pairs coregulating in liver (96 nonredundant genes). Two-hundred-and-fifty liver-specific genes were retrieved from the TiSGeD database that included human liver, fetal liver, hepatoma, and HepG2 specific genes. In total, 542 liver-specific genes were retrieved from these resources.

Liver-specific target genes were identified for the microRNAs of interest by comparing all the target genes with four liver-specific gene lists based on TiGER (EST, CRM, TF) and TiSGeD. A target gene was deemed to be liver-specific if it was present in any of the four liver-specific gene lists.

### 2.10. Validation of Candidate MicroRNAs

Candidate microRNAs targeting liver-specific genes were selected for further validation by quantitative real-time PCR (qRT-PCR). Candidate microRNAs were individually quantified by standard qRT-PCR using total RNA extracted from plasma of 34 HBeAg-positive, 26 HBeAg-negative, and 60 healthy controls.

### 2.11. RNA Extraction

Total RNA was extracted from plasma using the miRNeasy mini kit (Qiagen, Hilden, Germany) as previously described [4]. RNA was stored at −80°C until further use.

### 2.12. cDNA Synthesis

cDNA synthesis was performed using the Universal cDNA Synthesis kit (Exiqon, Vedbaek, Denmark) as previously described [4] and cDNA was stored at −20°C.

### 2.13. qRT-PCR

qRT-PCRs were performed on Pick & Mix microRNA PCR panels including predesigned primers for 23 microRNAs: mir-16-2-3p, -28-5p, -30a-5p, -30b-5p, -30c-5p, -30e-3p, -144-5p, -148a-3p, -224-3p, -378a-3p, -548c-5p, -574-3p, -589-3p, -605, -636, -639, -654-3p, -let-7c, -23a-3p, -451a, 22-5p, -26a-5p, 221-3p (Exiqon, Vedbaek, Denmark). Resultant cDNA was diluted ×50 and assayed in 10 *μ*L PCR reactions according to the protocol for miRCURY LNA Universal RT microRNA PCR (Exiqon, Vedbaek, Denmark). Negative controls with no template from the reverse transcription reaction were included and profiled like the samples. Thermal cycling was performed on a CFX384 Real-Time thermal cycler (Biorad, Hercules, California, USA) in 384 well plates as per Exiqon's instruction. *C*_*T*_ (max) was set to 40 amplification cycles. Analyses were run in triplicate.

### 2.14. Analysis of qRT-PCR Data

Quality control of the qRT-PCR was performed as previously described [4, 20]. Raw data were normalised using the geometric mean of miR-22-3p, -26a-5p, and -221-3p [4, 21, 22] and the comparative *C*_*T*_ method was used to analyse the data [16]. Statistical analyses were performed as previously described [4], by using SAS software, version 9.2 (SAS Institute, Cary, NC, USA). Briefly, statistical significances were determined using Mann-Whitney test and correlation analyses were performed in two steps, both of which used analysis of variance on ranks (*P* values from Chi-Squared tests). Due to multiple testing only *P* < 0.0028 was regarded as significant (Bonferroni correction).

## 3. Results

### 3.1. Clinical Parameters

HBsAg was quantified in plasma from 48 out of 60 (80%) children with CHB. Plasma levels of HBsAg were significantly higher in HBeAg-positive children (1.1*E* + 08 ± SD  9.3*E* + 07) compared to HBeAg-negative children (1.4*E* + 07 ± SD  1.2*E* + 07), *P* < 0.001. It was not possible to measure HBsAg in plasma from 12 chronically infected children due to lack of sample material (7 HBeAg-positive and 5 HBeAg-negative).

### 3.2. Selection of Candidate MicroRNAs

We revisited our previous screen on plasma levels of 739 microRNAs in HBeAg-positive, HBeAg-negative, and healthy children [4]. Only data from children with CHB were included in the present study. Firstly, we included only microRNAs with raw *C*_*T*_-values at or below 35 for further downstream analyses. Based on this cut-off, 227 candidate microRNAs were taken for subsequent analysis. Secondly, raw data of the 227 candidate microRNAs were normalised against the global mean; U6; and geometric mean of miR-22-5p, -26a-5p, and -221-3p. The plasma microRNAs levels in HBeAg-positive and HBeAg-negative children were compared and the top up- and downregulated microRNAs were selected. A total of 32 microRNAs were selected as candidate microRNAs. The candidate microRNAs included 14 microRNAs that were previously identified (miR-99a-5p, -100-5p, -122-5p, -122-3p, -125b-5p, -192-5p, -192-3p, -193b-3p, -194-5p, -215, -365a-3p, -455-5p, -455-3p, and -855-5p) [4] and 18 “new” candidate microRNAs of which 9 showed higher levels in plasma from HBeAg-positive children compared to HBeAg-negative children (miR-28-5p, -30a-5p, -30b-5p, -30c-5p, -30e-3p, -148a-3p, -378a-3p, -574-3p, and -let-7c) and nine showed lower levels (miR-16-2-3p, -144-5p, -224-3p, -548c-5p, -589-3p, -605, -636, -639, and 654-3p).

### 3.3. Identification of Liver-Specific MicroRNA Target Genes

All 32-candidate microRNAs were included in bioinformatics analysis. MicroRNA target genes with CLIP-seq overlap were retrieved from the starBase, 26 microRNAs (miR-28-5p, -30a-5p, -30b-5p, -30c-5p, -30e-3p, -99a-5p, -100-5p, -122-5p, -125b-5p, -148a-3p, -192-5p, -193b-3p, -194-5p, -215, -365a-3p, -378a-3p, -455-5p, -455-3p, -548c-5p, -574-3p, -605, -636, -639, -654-3p, -855-5p, and -let-7c) had target genes with CLIP-seq overlap, and a total of 1822 target genes with CLIP-seq overlap were retrieved from the starBase.

A liver-specificity filter was applied on the retrieved microRNA target genes with CLIP-seq-overlap. Sixteen out of 26 microRNAs had liver-specific target genes with CLIP-seq overlap (miR-28-5p, -30a-5p, -30e-3p, -125b-5p, -148a-3p, -193b-3p, -215, -365a-3p, -378a-3p, -455-5p, -455-3p, -548c-5p, -574-3p, -639, -654-3p, and -let-7c), and in total 28 (16 non-redundant) target genes were retrieved. The 16 non-redundant target genes were* ACADSB, ARID1A, BTG3, CEBPG, CPOX, E2F1, FRAT2, GABBR1, GATA6, HOXA9, LEF1, MAZ, PAPD5, SF1, SMAD4*, and* ZXDB* (Table 2).

Furthermore, the remaining ten microRNAs had no liver-specific target genes and did not pass the liver-specificity filter. For these microRNAs, the total numbers of non-liver-specific target genes with CLIP-seq overlap retrieved from the starBase were 1378 (833 nonredundant). Only those target genes predicted by two or more target prediction tools are presented: in total 522 target genes of which 299 are nonredundant (Table S1 in Supplementary Material, available online at http://dx.doi.org/10.1155/2014/791045).

### 3.4. Validation of Candidate MicroRNAs

Of the 16 candidate microRNAs targeting liver-specific genes, six microRNAs (miR-125b-5p, -193b-3p, -215, -365a-3p, -455-5p, and -455-3p) were validated in our previous study. Interestingly, all these microRNAs were significantly expressed at higher levels in plasma from HBeAg-positive children than in the HBeAg-negative children [4]. The remaining 10 microRNAs were selected for validation in the present study (miR-28-5p, -30a-5p, -30e-3p, -148a-3p, -378a-3p, -548c-5p, -574-3p, -639, -654-3p, and -let-7c). Individual qRT-PCR analyses of these microRNAs were performed on plasma samples from 34 HBeAg-positive, 26 HBeAg-negative, and 60 healthy children. Quality control led to the exclusion of samples from four children (one sample from a HBeAg-negative child and three samples from healthy controls) and the exclusion of two microRNAs (miR-148a-3p and -639).

### 3.5. MicroRNAs Showing Aberrant Plasma Levels in HBeAg-Positive Children

**Figure 1:**
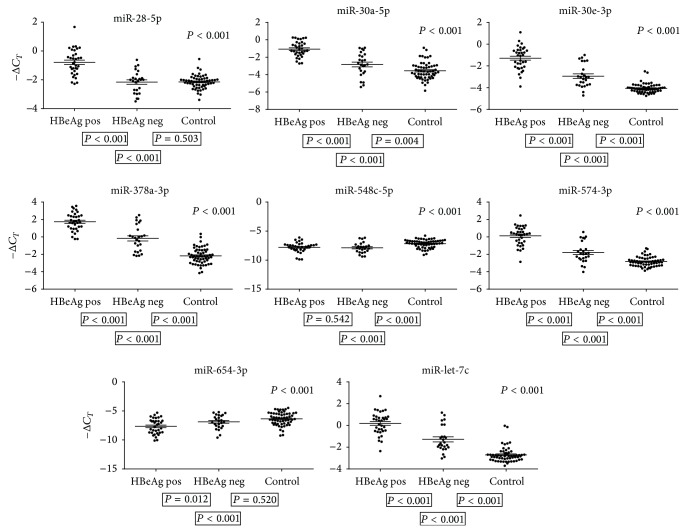
MicroRNA plasma levels in HBeAg-positive, HBeAg-negative, and healthy children. qRT-PCR-data on 34 HBeAg-positive, 25 HBeAg-negative, and 57 healthy control children. The *P* values shown above the individual figures are results of statistical analyses on HBeAg-positive, HBeAg-negative, and healthy children, and *P* values shown below left are results on HBeAg-positive versus HBeAg-negative, below centred HBeAg-positive versus healthy controls, and below right HBeAg-negative versus healthy controls. The bars represent geometric means −Δ*C*_*T*_ ± SEM. Due to multiple testing only *P* < 0.0028 was considered significant.

We measured the quantity of the eight microRNAs in plasma from 34 HBeAg-positive and 25 HBeAg-negative children. Six microRNAs (miR-28-5p, -30a-5p, -30e-3p, -378a-3p, -574-3p, and -let-7c) were significantly expressed (*P* < 0.001) at higher levels in plasma from HBeAg-positive children than in the HBeAg-negative children. One microRNA (miR-654-3p) was expressed at a lower level (*P* = 0.012) (Figure 1). Due to multiple testing, however, only *P* < 0.0028 was regarded as significant. miR-548c-5p was expressed at the same level in plasma from HBeAg-positive and HBeAg-negative children, *P* = 0.542 (Figure 1).

To further investigate the microRNA expression patterns of children with CHB we included data from 57 healthy controls in the statistical analyses. Of the six microRNAs with significantly higher plasma levels in HBeAg-positive children than in HBeAg-negative children, two microRNAs (miR-28-5p and miR-30a-5p) showed similar expression levels in plasma from HBeAg-negative and healthy children, *P* > 0.004, whereas four microRNAs (miR-30e-3p, 378a-3p, 574-3p, and let-7c) showed lowest expression in plasma from healthy controls, *P* < 0.001 (Figure 1).

miR-654-3p that showed lower expression in HBeAg-positive children compared to HBeAg-negative children was expressed at same levels in plasma from HBeAg-negative and healthy control children, *P* = 0.52 (Figure 1).

Levels (fold changes) of the eight microRNAs in plasma from HBeAg-positive and HBeAg-negative children were calculated relative to the plasma levels in healthy controls (Table 3).

### 3.6. MicroRNA Plasma Levels and Virological Parameters

We examined the correlations between plasma microRNA levels and the virological parameters: viral load and HBsAg quantity. Only children with CHB were included in these analyses. Firstly, focusing on univariate analyses, we observed strong positive correlations between plasma levels of six microRNAs (miR-28-5p, -30a-5p, -30e-3p, -378a-3p, -574-3p, and -let-7c) and with both viral load and HBsAg quantity, *P* < 0.001. All six of these microRNAs showed significantly higher plasma levels in HBeAg-positive children when compared to HBeAg-negative children.

**Figure 2:**
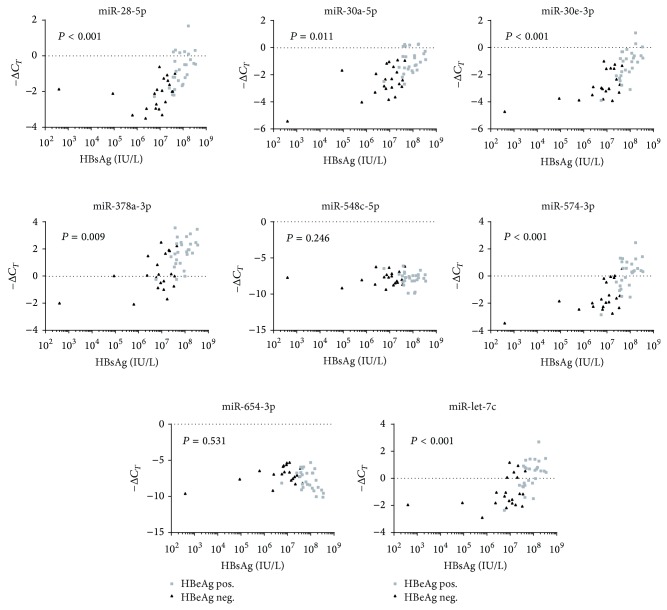
MicroRNA plasma levels and HBsAg quantity. qRT-PCR and HBsAg data on 34 HBeAg-positive and 25 HBeAg-negative children. *P* values are based on multivariate analyses. Due to multiple testing only *P* < 0.0028 was considered significant.

Secondly, multivariate analyses were performed taking into account the effect on plasma microRNA levels of the different virological parameters: HBeAg-status, viral load, and HBsAg quantity. Interestingly, the strongest correlations were observed between plasma microRNA levels and HBsAg quantity. The correlations were significant for four microRNAs (miR-28-5p, -30e-3p, -574-3p, and -let-7c), *P* < 0.001, all of which showed significantly higher levels in HBeAg-positive children when compared to HBeAg-negative children (Figure 2).

## 4. Discussion

HBeAg-positive children are especially vulnerable to developing HCC and cirrhosis, and we hypothesise that specific microRNAs impact the progression of CHB. This study is the first to identify microRNAs with aberrant plasma expressions specifically in HBeAg-positive children and with liver-specific target genes.

A total of 13 microRNAs showed aberrant plasma expressions in HBeAg-positive children and revealed liver-specific target genes (miR-28-5p, -30a-5p, -30e-3p, -125b-5p, -193b-3p, -215, -365a-3p, -378a-3p, -455-5p, -455-3p, -574-3p, -654-3p, and -let-7c). Our bioinformatics approach led to the identification of 21 liver-specific target genes for the 13 microRNAs. Of these, 13 were nonredundant. All target genes retrieved for two or more microRNAs (*ARIDIA, CEBPG, HOXA9*, and* MAZ*), and the majority of the remaining target genes (*CPOX, E2F1, FRAT2, GATA6, SF1*, and* SMAD4*) are regulators of carcinogenesis, functioning both as promoters and as suppressors, and/or are implicated in the replication of HBV [23–34]. However, three of the target genes, to our knowledge, are not described in relation to cancer or HBV infection (*ACADSB, GABBR1*, and* PAPD5*).

For miR-122, known to be highly expressed in liver tissue [35], we could not identify liver-specific target genes due to nonavailability of CLIP-seq data for a number of microRNAs, including mir-122-3p. Additionally, the application of a liver-specificity filter excluded candidate microRNAs with substantial functions in tissues other than the liver.

Of the 13 microRNAs targeting liver-specific genes, seven microRNAs (validated in the present study) were aberrantly expressed in HBeAg-positive children when compared to HBeAg-negative children. Six microRNAs (miR-28-5p, -30a-5p, -30e-3p, -378a-3p, -574-3p, and -let-7c) showed significantly higher levels in HBeAg-positive children when compared to HBeAg-negative children, and one microRNA (miR-654-3p) showed lower levels (*P* = 0.012). Additionally, six out of the 13 microRNAs with liver-specific target genes were validated in our previous study. All six microRNAs (miR-125b-5p, -193b-3p, -215, -365a-3p, -455-5p, and -455-3p) [4] showed significantly higher levels in HBeAg-positive children when compared to HBeAg-negative children. Interestingly, all 12 microRNAs upregulated in HBeAg-positive children are previously reported aberrantly expressed in adults with different types of cancer [36–44]. Indeed, seven microRNAs (miR-30a-5p, -125b-5p, -193b-3p, -215, -378a-3p, -574-3p, and -let-7c) have previously been associated with HCC [39–43, 45, 46].

By including data on healthy controls in the analyses we identified two interesting microRNA expression profiles. Firstly, three microRNAs (miR-28-5p, -30a-5p, and -125b-5p) were significantly upregulated in HBeAg-positive children when compared to HBeAg-negative and healthy control children, which showed equal levels. The liver-specific target genes identified for these three microRNAs were* ARID1A, FRAT2*, and* MAZ*.* ARID1A* encodes an AT-rich interactive domain-containing protein 1A (SWI-like).* ARID1A* is a tumour suppressor and has been found mutated in HBV associated HCC [24, 47].* FRAT2* encodes a GSK-3-binding protein, and the expression of* FRAT2* may be upregulated in tumour progression [25].* MAZ* gene encodes a MYC-associated zinc finger protein and the expression of MAZ is significantly increased in HCC [26]. Interestingly, the signalling pathways regulated by* ARID1A, FRAT2*, and* MAZ* have at least one target in common, namely, c-*MYC* [48–50], which is known to be overexpressed in mouse HCC [51].

The other interesting microRNA expression profile observed in the present study was for miR-654-3p. miR-654-3p was downregulated in HBeAg-positive children when compared to HBeAg-negative and healthy control children, which showed equal levels. In line with our finding, miR-654-3p has an inhibitory role in the H1N1 Influenza A virus [52] and acts as a tumor suppressor in prostate cancer [53]. Two liver-specific target genes,* CPOX* and* SF1*, are targeted by miR-654-3p.* CPOX* encodes the enzyme Coproporphyrinogen-III oxidase that has a role in the haem and chlorophyll biosynthetic pathways [54] whereas* SF1* encodes Splicing Factor 1 that is involved in the formation of the spliceosome complex [55].

We propose that these microRNAs identified with aberrant plasma expressions specifically in HBeAg-positive children and with liver-specific target genes are biomarkers for disease progression and might impact the development of HCC, and perhaps also cirrhosis, in children with CHB. Functional studies are warranted, however, to further elucidate these microRNAs' role in the immunopathogenesis of childhood CHB.

To date few therapies have been licensed to treat CHB in children, and most are only partially effective [5]. Interestingly, the potential of microRNAs as targets for therapeutic interventions has shown great promise [11], and the first artificial microRNA antagonist is already in clinical trial [56]. MicroRNAs with a biological function in the immunopathogenesis of childhood CHB might serve as targets for the development of new antiviral treatment strategies. Whether the microRNAs identified in the present study would have the potential to serve as targets in future therapeutics needs to be investigated.

In conclusion, we identified 13 microRNAs with aberrant plasma expressions of HBeAg-positive children and with liver-specific target genes. In particular, we observed two distinct microRNA expression patterns: three microRNAs were upregulated and one was downregulated in HBeAg-positive children compared to HBeAg-negative and healthy control children, which showed equal levels. Five liver-specific target genes were identified for the four microRNAs. The microRNAs might be biomarkers for disease progression in children with CHB. Further studies are needed to elucidate the microRNAs' role in childhood CHB, hopefully leading to the identification of future therapeutic targets and to enhanced management of childhood CHB.

## Supplementary Material

Ten candidate microRNAs had no liver specific target genes. For these ten microRNAs, the total numbers of non-liver-specific target genes with CLIP-seq overlap retrieved from the Starbase were 1378. Here, only those target genes predicted by two or more target prediction tools are presented: in total 522.

## Figures and Tables

**Table 1:** Characteristics of children with chronic hepatitis B and of healthy controls [4].

	Chronic hepatitis B		Healthy controls
	HBeAg pos.	HBeAg neg.	
Number of patients	**34**	**26**	**60**
Male	14 (41%)	12 (46%)	33 (55%)
Female	20 (59%)	14 (54%)	27 (45%)
Age (years)			
Mean	**8.8**	**12.0**	**7.1**
SD	3.7	3.4	3.7
Race			
African	4	6	1
Asian	22	15	9
Caucasian	8	5	50
ALT (U/L ref. value 5–45)			
Mean	**46.5**	**25.6**	**14.3**
SD	27.6	12.6	5.3
HBV DNA (IU/mL)			
Mean	**5.1*E *+* *08**	**8.5*E *+* *02**	**NA**
SD	1.2*E* + 09	2.0*E* + 03	
Genotypes			
A	**2** (6%)	**2** (8%)	
B	**11** (32%)	**2** (8%)	
C	**5** (15%)	**2** (8%)	
D	**12** (35%)	**7** (27%)	
E	**3** (9%)	**2** (8%)	
F	**1** (3%)	**0** (0%)	
N/A	**0** (0%)	**11** (42%)	**60** (100%)

ALT: alanine aminotransferase.

N/A: not analysed.

**Table 2:** Liver-specific target genes with CLIP-seq overlap for 16 microRNAs.

MicroRNA (hsa)	Target gene	Gene description
28-5p	MAZ	MYC-associated zinc finger protein (purine-binding transcription factor)

30a-5p	ARID1A	AT rich interactive domain 1A (SWI-like)

30e-3p	CEBPG	CCAAT/enhancer binding protein (C/EBP), gamma

125b-5p	FRAT2	Frequently rearranged in advanced T-cell lymphomas 2
	MAZ	MYC-associated zinc finger protein (purine-binding transcription factor)

148a-3p	ZXDB	Zinc finger, X-linked, duplicated B
	ARID1A	AT rich interactive domain 1A (SWI-like)
	CEBPG	CCAAT/enhancer binding protein (C/EBP), gamma

193b-3p	MAZ	MYC-associated zinc finger protein (purine-binding transcription factor)

215	ACADSB	Acyl-CoA dehydrogenase, short/branched chain

365a-3p	HOXA9	Homeobox A9
	MAZ	MYC-associated zinc finger protein (purine-binding transcription factor)

378a-3p	ARID1A	AT rich interactive domain 1A (SWI-like)

455-5p	SMAD4	SMAD family member 4

455-3p	PAPD5	PAP associated domain containing 5
	MAZ	MYC-associated zinc finger protein (purine-binding transcription factor)

548c-5p	BTG3	B-cell translocation gene 3
	SF1	Splicing factor 1
	GATA6	GATA binding protein 6

574-3p	GABBR1	Gamma-aminobutyric acid (GABA) B receptor, 1
	E2F1	E2F transcription factor 1

639	LEF1	Lymphoid enhancer-binding factor 1

654-3p	CPOX	Coproporphyrinogen oxidase
	SF1	Splicing factor 1

let-7c	HOXA9	Homeobox A9
	GATA6	GATA binding protein 6
	CEBPG	CCAAT/enhancer binding protein (C/EBP), gamma
	SF1	Splicing factor 1

**Table 3:** MicroRNA plasma levels in children with CHB relative to healthy controls.

MicroRNA (hsa)	Sample	Mean Ct	SD	Fold change	SE	*P* value
28-5p	HBeAg pos.	28.21	1.35	3.1	2.8–3.5	<0.001
	HBeAg neg.	29.89	1.80	1.0	0.9–1.1	
	Control	29.39	1.34	1		

30a-5p	HBeAg pos.	28.50	1.22	5.6	4.9–6.4	<0.001
	HBeAg neg.	30.55	1.07	1.6	1.3–2.0	
	Control	30.80	0.65	1		

30e-3p	HBeAg pos.	28.72	1.60	6.8	6.0–7.8	<0.001
	HBeAg neg.	30.77	1.36	2.9	2.5–3.3	
	Control	31.34	1.25	1		

378a-3p	HBeAg pos.	25.69	1.39	15.1	13.0–17.5	<0.001
	HBeAg neg.	27.88	0.93	4.0	3.3–5.0	
	Control	29.43	0.71	1		

548c-5p	HBeAg pos.	35.17	1.57	0.7	0.6-0.7	<0.001
	HBeAg neg.	35.43	1.94	0.9	0.8–1.0	
	Control	34.40	1.46	1		

574-3p	HBeAg pos.	27.30	1.48	7.8	6.8–9.0	<0.001
	HBeAg neg.	29.51	1.22	2.1	1.7–2.4	
	Control	30.07	0.98	1		

654-3p	HBeAg pos.	35.11	1.82	0.4	0.3–0.5	<0.001
	HBeAg neg.	34.68	2.16	0.7	0.6–0.8	
	Control	33.62	1.94	1		

let-7c	HBeAg pos.	27.24	1.53	7.4	6.5–8.5	<0.001
	HBeAg neg.	29.02	1.36	2.7	2.3–3.2	
	Control	29.94	1.15	1		
