# Supplementary material for: Circulating MicroRNAs in Plasma of Hepatitis B e Antigen Positive Children Reveal Liver-Specific Target Genes
Source: Int J Hepatol. 2014 Dec 17;2014:791045. doi: 10.1155/2014/791045 (PMC4281389; doi:10.1155/2014/791045)
Supplement: Supplementary file 1 — Ten candidate microRNAs had no liver specific target genes. For these ten microRNAs, the total numbers of non-liver-specific target genes with CLIP-seq overlap retrieved from the Starbase were 1378. Here, only those target genes predicted by two or more target prediction tools are presented: in total 522. [file 791045.f1.pdf]

**Table S1. Non-liver-specific target genes with CLIP-seq overlap for 10 microRNAs predicted by two or more tools.**

| MicroRNA (hsa) | GeneName | refGene | TargetScan | picTar | RNA22 | PITA | miRanda |
|---|---|---|---|---|---|---|---|
| 30b-5p | PPTC7 | NM_139283 | 3 | 3 | 0 | 3 | 0 |
| | SOX4 | NM_003107 | 31 | 31 | 0 | 31 | 73 |
| | NDEL1 | NM_001025579 | 86 | 0 | 0 | 86 | 86 |
| | KLF9 | NM_001206 | 0 | 1 | 0 | 1 | 10 |
| | QKI | NM_206853 | 218 | 0 | 0 | 218 | 0 |
| | FBXO45 | NM_001105573 | 7 | 0 | 0 | 7 | 0 |
| | AZIN1 | NM_148174 | 26 | 26 | 0 | 0 | 0 |
| | SIX4 | NM_017420 | 31 | 0 | 0 | 31 | 31 |
| | TFDP1 | NR_026580 | 7 | 0 | 0 | 7 | 0 |
| | RAB8A | NM_005370 | 4 | 4 | 0 | 4 | 4 |
| | C13orf37 | NM_001071775 | 65 | 0 | 0 | 61 | 65 |
| | LIFR | NM_001127671 | 148 | 0 | 0 | 148 | 0 |
| | LRRC8D | NM_001134479 | 3 | 0 | 0 | 3 | 0 |
| | MLL3 | NM_170606 | 2 | 0 | 0 | 2 | 2 |
| | B3GNT5 | NM_032047 | 5 | 5 | 0 | 5 | 7 |
| | JAK1 | NM_002227 | 8 | 0 | 0 | 8 | 8 |
| | SATB1 | NM_002971 | 3 | 3 | 0 | 0 | 0 |
| | SNIP1 | NM_024700 | 0 | 0 | 2 | 0 | 2 |
| | UBN2 | NM_173569 | 19 | 0 | 0 | 19 | 0 |
| | OTUD4 | NM_001102653 | 7 | 0 | 0 | 7 | 0 |
| | FOXG1 | NM_005249 | 6 | 6 | 0 | 6 | 6 |
| | CCDC43 | NM_001099225 | 6 | 0 | 0 | 6 | 0 |
| | CELSR3 | NM_001407 | 7 | 7 | 0 | 7 | 14 |
| | PSMD7 | NM_002811 | 35 | 35 | 0 | 35 | 35 |
| | SLC38A2 | NM_018976 | 2 | 2 | 0 | 2 | 2 |
| | PTP4A1 | NM_003463 | 12 | 12 | 0 | 12 | 0 |
| | WDR82 | NM_025222 | 7 | 0 | 0 | 7 | 0 |
| | CHMP2B | NM_014043 | 13 | 13 | 0 | 13 | 0 |
| | GJA1 | NM_000165 | 5 | 5 | 0 | 5 | 5 |
| | ZNF280B | NM_080764 | 8 | 8 | 0 | 8 | 8 |
| | SP4 | NM_003112 | 13 | 0 | 0 | 13 | 13 |
| | TAOK1 | NM_020791 | 24 | 24 | 0 | 24 | 24 |
| | NFYB | NM_006166 | 2 | 0 | 0 | 2 | 2 |
| | CPEB3 | NM_014912 | 3 | 3 | 0 | 3 | 3 |
| | E2F3 | NM_001949 | 0 | 0 | 4 | 0 | 4 |
| | COPS7B | NM_022730 | 25 | 25 | 0 | 25 | 25 |
| | SFRS7 | NM_001031684 | 28 | 28 | 0 | 28 | 28 |
| | SEMA6D | NM_153618 | 2 | 2 | 0 | 2 | 2 |
| | TULP4 | NM_020245 | 0 | 7 | 0 | 0 | 5 |
| | PCGF5 | NM_032373 | 6 | 6 | 0 | 6 | 6 |
| | OTUD4 | NM_199324 | 0 | 7 | 0 | 0 | 7 |
| | BACH1 | NM_206866 | 16 | 16 | 0 | 0 | 0 |
| | RAP2C | NM_021183 | 7 | 7 | 0 | 7 | 7 |
| | ARL6IP6 | NR_024526 | 5 | 0 | 6 | 5 | 0 |
| | CCDC43 | NM_144609 | 0 | 6 | 0 | 0 | 6 |
| | PPP3R1 | NM_000945 | 6 | 6 | 0 | 6 | 6 |

| | | | | | | | |
|---|---|---|---|---|---|---|---|
| | PIP4K2B | NM_003559 | 4 | 0 | 0 | 4 | 4 |
| | ZNF238 | NM_205768 | 15 | 0 | 0 | 15 | 15 |
| | CALU | NM_001130674 | 4 | 0 | 0 | 4 | 0 |
| | RARB | NM_000965 | 0 | 16 | 0 | 0 | 16 |
| | EDC3 | NM_001142443 | 7 | 0 | 0 | 7 | 0 |
| | CBFB | NM_001755 | 0 | 2 | 0 | 0 | 2 |
| | SCAMP1 | NM_004866 | 9 | 9 | 0 | 9 | 9 |
| | TBPL1 | NM_004865 | 2 | 2 | 0 | 0 | 0 |
| | FLJ36031 | NM_175884 | 4 | 4 | 0 | 4 | 4 |
| | GMEB2 | NM_012384 | 11 | 11 | 0 | 11 | 11 |
| | ZSCAN29 | NM_152455 | 2 | 0 | 0 | 2 | 2 |
| | ZNF507 | NM_014910 | 20 | 0 | 0 | 20 | 20 |
| | YPEL5 | NM_016061 | 0 | 4 | 0 | 0 | 4 |
| | CBX3 | NM_016587 | 11 | 0 | 0 | 11 | 0 |
| | SETD5 | NM_001080517 | 7 | 0 | 0 | 7 | 8 |
| | EML4 | NM_001145076 | 10 | 0 | 0 | 10 | 0 |
| | PLAGL2 | NM_002657 | 6 | 0 | 0 | 6 | 6 |
| | TMED2 | NM_006815 | 0 | 7 | 0 | 7 | 7 |
| | KLHL28 | NM_017658 | 10 | 0 | 0 | 10 | 81 |
| | GFPT2 | NM_005110 | 7 | 7 | 0 | 7 | 7 |
| | KRAS | NM_033360 | 0 | 9 | 0 | 9 | 0 |
| | PLEKHO2 | NM_025201 | 2 | 0 | 0 | 2 | 2 |
| | ARID3A | NM_005224 | 3 | 3 | 0 | 0 | 3 |
| | C13orf23 | NM_025138 | 12 | 12 | 12 | 12 | 12 |
| | TXNDC12 | NM_015913 | 0 | 0 | 11 | 0 | 11 |
| | ZBTB41 | NM_194314 | 9 | 0 | 0 | 9 | 9 |
| | MKRN3 | NM_005664 | 34 | 34 | 0 | 34 | 34 |
| | FRS2 | NM_006654 | 8 | 0 | 0 | 8 | 8 |
| | IDH1 | NM_005896 | 10 | 10 | 0 | 0 | 0 |
| | SEC24A | NM_021982 | 0 | 0 | 7 | 7 | 7 |
| | RNF44 | NM_014901 | 3 | 3 | 0 | 3 | 3 |
| | TULP4 | NM_001007466 | 5 | 0 | 0 | 5 | 0 |
| | AP2A1 | NM_014203 | 0 | 9 | 0 | 0 | 9 |
| | EED | NM_003797 | 12 | 12 | 0 | 12 | 6 |
| | SLC6A6 | NM_003043 | 14 | 14 | 0 | 7 | 7 |
| | C10orf26 | NM_001083913 | 2 | 0 | 0 | 2 | 0 |
| | FAM91A1 | NM_144963 | 1 | 0 | 0 | 1 | 1 |
| | KIAA0247 | NM_014734 | 3 | 0 | 0 | 3 | 3 |
| | FAM160B1 | NM_020940 | 3 | 0 | 3 | 0 | 3 |
| | DHX40 | NM_001166301 | 2 | 0 | 0 | 2 | 0 |
| | LCLAT1 | NM_182551 | 0 | 5 | 0 | 0 | 5 |
| | RARB | NM_016152 | 16 | 16 | 0 | 16 | 0 |
| | PRPF40A | NM_017892 | 10 | 0 | 0 | 10 | 0 |
| | GLCE | NM_015554 | 1 | 7 | 0 | 7 | 1 |
| | CALD1 | NM_033138 | 2 | 0 | 0 | 2 | 2 |
| | PSME3 | NM_176863 | 6 | 6 | 0 | 6 | 6 |
| | RAP2B | NM_002886 | 14 | 0 | 0 | 14 | 14 |
| | E2F7 | NM_203394 | 110 | 110 | 0 | 0 | 0 |
| | SH2B3 | NM_005475 | 11 | 0 | 0 | 11 | 0 |
| | ERLIN1 | NM_006459 | 9 | 9 | 0 | 9 | 9 |
| | KIAA1211 | NM_020722 | 3 | 0 | 0 | 3 | 0 |

| | | | | | | |
|---|---|---|---|---|---|---|
| XPO1 | NM_003400 | 14 | 14 | 0 | 14 | 14 |
| TMEM87A | NM_015497 | 4 | 4 | 0 | 4 | 4 |
| STK39 | NM_013233 | 2 | 2 | 0 | 2 | 2 |
| CCNT2 | NM_058241 | 14 | 14 | 0 | 0 | 0 |
| NAA25 | NM_024953 | 2 | 12 | 0 | 2 | 12 |
| NRBP1 | NM_013392 | 0 | 0 | 0 | 1 | 1 |
| CALU | NM_001219 | 0 | 4 | 0 | 0 | 5 |
| S100PBP | NM_022753 | 7 | 0 | 0 | 0 | 7 |
| ARL6IP6 | NM_152522 | 0 | 5 | 0 | 0 | 6 |
| PHACTR2 | NM_001100166 | 10 | 0 | 0 | 10 | 0 |
| SYPL1 | NM_182715 | 8 | 8 | 0 | 8 | 8 |
| RAB23 | NM_183227 | 6 | 0 | 0 | 6 | 0 |
| PPP1R14C | NM_030949 | 2 | 2 | 0 | 2 | 2 |
| TMEM33 | NM_018126 | 0 | 0 | 0 | 3 | 3 |
| PHF16 | NM_014735 | 5 | 5 | 0 | 5 | 0 |
| DLG5 | NM_004747 | 2 | 2 | 0 | 0 | 0 |
| TGDS | NM_014305 | 0 | 0 | 0 | 5 | 5 |
| RFX7 | NM_022841 | 1 | 0 | 0 | 1 | 1 |
| SNX10 | NM_013322 | 1 | 0 | 0 | 0 | 1 |
| UBE3C | NM_014671 | 3 | 0 | 0 | 3 | 0 |
| BCOR | NM_001123385 | 48 | 0 | 0 | 48 | 0 |
| BDP1 | NM_018429 | 7 | 0 | 0 | 0 | 7 |
| TNRC6A | NM_014494 | 8 | 6 | 0 | 8 | 8 |
| ELMOD2 | NM_153702 | 3 | 3 | 0 | 3 | 3 |
| AP2A1 | NM_130787 | 9 | 9 | 0 | 9 | 0 |
| KPNA6 | NM_012316 | 3 | 0 | 0 | 3 | 0 |
| PFN2 | NM_002628 | 9 | 9 | 0 | 9 | 0 |
| UBE2V2 | NM_003350 | 3 | 3 | 0 | 3 | 3 |
| DDAH1 | NM_012137 | 24 | 24 | 0 | 24 | 24 |
| ODZ3 | NM_001080477 | 9 | 0 | 0 | 9 | 9 |
| MIER3 | NM_152622 | 73 | 94 | 0 | 90 | 90 |
| DDIT4 | NM_019058 | 1 | 1 | 0 | 1 | 1 |
| LMBR1L | NM_018113 | 60 | 60 | 0 | 60 | 120 |
| SERPINE1 | NM_000602 | 1 | 1 | 0 | 1 | 0 |
| ELL | NM_006532 | 2 | 2 | 0 | 2 | 2 |
| NEFL | NM_006158 | 1 | 0 | 0 | 1 | 1 |
| DHX40 | NM_024612 | 0 | 2 | 0 | 0 | 2 |
| LIN28B | NM_001004317 | 10 | 0 | 0 | 10 | 10 |
| REEP3 | NM_001001330 | 19 | 19 | 0 | 19 | 132 |
| GLCCI1 | NM_138426 | 12 | 0 | 0 | 12 | 12 |
| ZDHHC21 | NM_178566 | 8 | 8 | 0 | 8 | 8 |
| NRBF2 | NM_030759 | 1 | 1 | 0 | 0 | 0 |
| PFN2 | NM_053024 | 0 | 9 | 0 | 0 | 9 |
| ZFX | NM_003410 | 0 | 0 | 0 | 3 | 3 |
| GOLGA8B | NM_001023567 | 0 | 0 | 0 | 2 | 2 |
| NUFIP2 | NM_020772 | 82 | 82 | 0 | 82 | 82 |
| PRKRIR | NM_004705 | 2 | 0 | 0 | 2 | 2 |
| C1orf135 | NM_024037 | 1 | 0 | 0 | 0 | 1 |
| FRZB | NM_001463 | 8 | 0 | 0 | 8 | 8 |
| ELOVL5 | NM_021814 | 5 | 5 | 0 | 5 | 5 |
| RAPH1 | NM_213589 | 5 | 5 | 0 | 0 | 5 |

| | | | | | | | |
|---|---|---|---|---|---|---|---|
| | ZNF711 | NM_021998 | 92 | 0 | 0 | 3 | 3 |
| | BCOR | NM_017745 | 0 | 48 | 0 | 0 | 48 |
| | PDCD10 | NM_145859 | 7 | 7 | 0 | 0 | 0 |
| | GNPDA1 | NM_005471 | 9 | 0 | 0 | 9 | 9 |
| | PTGFRN | NM_020440 | 46 | 46 | 0 | 0 | 23 |
| | PIP4K2A | NM_005028 | 16 | 16 | 0 | 16 | 16 |
| | CBFB | NM_022845 | 2 | 2 | 0 | 2 | 0 |
| | CD2AP | NM_012120 | 11 | 0 | 0 | 11 | 11 |
| | SPEN | NM_015001 | 44 | 44 | 0 | 10 | 10 |
| | PAWR | NM_002583 | 16 | 16 | 0 | 16 | 16 |
| | KLF10 | NM_005655 | 14 | 14 | 0 | 14 | 0 |
| | CEP350 | NM_014810 | 3 | 0 | 0 | 3 | 3 |
| | IGF1R | NM_000875 | 0 | 0 | 0 | 4 | 4 |
| | RAB11A | NM_004663 | 0 | 5 | 0 | 5 | 5 |
| | ZBTB39 | NM_014830 | 29 | 0 | 0 | 29 | 24 |
| | SLC16A14 | NM_152527 | 2 | 0 | 0 | 0 | 2 |
| | SATB2 | NM_015265 | 0 | 2 | 0 | 0 | 8 |
| | FAM104A | NM_032837 | 21 | 0 | 0 | 21 | 21 |
| | LCLAT1 | NM_001002257 | 5 | 5 | 0 | 5 | 0 |
| | YTHDF3 | NM_152758 | 43 | 43 | 0 | 43 | 43 |
| | EIF2C1 | NM_012199 | 3 | 3 | 0 | 3 | 3 |
| | LRRC8D | NM_018103 | 0 | 3 | 0 | 0 | 3 |
| | MAF | NM_001031804 | 4 | 0 | 0 | 4 | 4 |
| | GOLGA4 | NM_001172713 | 49 | 0 | 0 | 49 | 0 |
| | TXNDC5 | NM_001145549 | 17 | 0 | 0 | 17 | 0 |
| | HIC2 | NM_015094 | 46 | 46 | 0 | 46 | 46 |
| | CHD1 | NM_001270 | 26 | 0 | 0 | 26 | 26 |
| | PHF13 | NM_153812 | 18 | 18 | 0 | 0 | 0 |
| | CLTC | NM_004859 | 0 | 0 | 0 | 5 | 5 |
| | GNA13 | NM_006572 | 40 | 40 | 0 | 0 | 0 |
| | TNRC6B | NM_001162501 | 11 | 0 | 0 | 11 | 0 |
| | FAM81A | NM_152450 | 3 | 0 | 0 | 3 | 3 |
| 30c-5p | ARL6IP6 | NR_024526 | 5 | 5 | 6 | 5 | 6 |
| | C13orf23 | NM_025138 | 12 | 12 | 13 | 12 | 13 |
| | SOX4 | NM_003107 | 31 | 31 | 0 | 31 | 31 |
| | NDEL1 | NM_001025579 | 86 | 86 | 0 | 86 | 86 |
| | RAB8A | NM_005370 | 4 | 4 | 0 | 4 | 4 |
| | LRRC8D | NM_001134479 | 3 | 3 | 0 | 3 | 3 |
| | B3GNT5 | NM_032047 | 5 | 5 | 0 | 5 | 5 |
| | OTUD4 | NM_001102653 | 7 | 7 | 0 | 7 | 7 |
| | FOXG1 | NM_005249 | 6 | 6 | 0 | 6 | 6 |
| | CCDC43 | NM_001099225 | 6 | 6 | 0 | 6 | 6 |
| | CELSR3 | NM_001407 | 7 | 7 | 0 | 7 | 7 |
| | PSMD7 | NM_002811 | 35 | 35 | 0 | 35 | 35 |
| | SLC38A2 | NM_018976 | 2 | 2 | 0 | 2 | 2 |
| | SEMA6D | NM_153618 | 2 | 2 | 0 | 2 | 2 |
| | GJA1 | NM_000165 | 5 | 5 | 0 | 5 | 5 |
| | ZNF280B | NM_080764 | 8 | 8 | 0 | 8 | 8 |
| | TAOK1 | NM_020791 | 24 | 24 | 0 | 24 | 24 |
| | CPEB3 | NM_014912 | 3 | 3 | 0 | 3 | 3 |
| | COPS7B | NM_022730 | 25 | 25 | 0 | 25 | 25 |

| | | | | | | |
|---|---|---|---|---|---|---|
| SFRS7 | NM_001031684 | 28 | 28 | 0 | 28 | 28 |
| TULP4 | NM_001007466 | 5 | 5 | 0 | 5 | 5 |
| PCGF5 | NM_032373 | 6 | 6 | 0 | 6 | 6 |
| RAP2C | NM_021183 | 7 | 7 | 0 | 7 | 7 |
| PPP3R1 | NM_000945 | 6 | 6 | 0 | 6 | 6 |
| CALU | NM_001130674 | 4 | 4 | 0 | 4 | 5 |
| RARB | NM_016152 | 16 | 16 | 0 | 16 | 16 |
| CBFB | NM_022845 | 2 | 2 | 0 | 2 | 2 |
| SCAMP1 | NM_004866 | 9 | 9 | 0 | 9 | 9 |
| FLJ36031 | NM_175884 | 4 | 4 | 0 | 4 | 4 |
| GMEB2 | NM_012384 | 11 | 11 | 0 | 11 | 11 |
| PHF16 | NM_014735 | 5 | 5 | 0 | 5 | 5 |
| GFPT2 | NM_005110 | 7 | 7 | 0 | 7 | 7 |
| EED | NM_003797 | 6 | 6 | 0 | 6 | 6 |
| MKRN3 | NM_005664 | 34 | 34 | 0 | 34 | 34 |
| KLF10 | NM_005655 | 14 | 14 | 0 | 14 | 55 |
| RNF44 | NM_014901 | 3 | 3 | 0 | 3 | 3 |
| AP2A1 | NM_130787 | 9 | 9 | 0 | 9 | 9 |
| SLC6A6 | NM_003043 | 7 | 7 | 0 | 7 | 7 |
| DHX40 | NM_001166301 | 2 | 2 | 0 | 2 | 2 |
| LCLAT1 | NM_001002257 | 5 | 5 | 0 | 5 | 5 |
| GLCE | NM_015554 | 1 | 1 | 0 | 1 | 1 |
| PSME3 | NM_176863 | 6 | 6 | 0 | 6 | 6 |
| ERLIN1 | NM_006459 | 9 | 9 | 0 | 9 | 9 |
| XPO1 | NM_003400 | 14 | 14 | 0 | 14 | 14 |
| TMEM87A | NM_015497 | 4 | 4 | 0 | 4 | 4 |
| STK39 | NM_013233 | 2 | 2 | 0 | 2 | 2 |
| NAA25 | NM_024953 | 2 | 2 | 0 | 2 | 2 |
| SYPL1 | NM_182715 | 8 | 8 | 0 | 8 | 8 |
| PPP1R14C | NM_030949 | 2 | 2 | 0 | 2 | 2 |
| BCOR | NM_001123385 | 48 | 48 | 0 | 48 | 48 |
| TNRC6A | NM_014494 | 2 | 2 | 0 | 2 | 2 |
| TNRC6A | NM_014494 | 4 | 4 | 0 | 4 | 4 |
| ELMOD2 | NM_153702 | 3 | 3 | 0 | 3 | 3 |
| PFN2 | NM_002628 | 9 | 9 | 0 | 9 | 9 |
| UBE2V2 | NM_003350 | 3 | 3 | 0 | 3 | 3 |
| DDAH1 | NM_012137 | 24 | 24 | 0 | 24 | 24 |
| MIER3 | NM_152622 | 73 | 73 | 0 | 73 | 73 |
| DDIT4 | NM_019058 | 1 | 1 | 0 | 1 | 1 |
| LMBR1L | NM_018113 | 60 | 60 | 0 | 60 | 60 |
| ELL | NM_006532 | 2 | 2 | 0 | 2 | 2 |
| REEP3 | NM_001001330 | 19 | 19 | 0 | 19 | 132 |
| ZDHHC21 | NM_178566 | 8 | 8 | 0 | 8 | 8 |
| NUFIP2 | NM_020772 | 82 | 82 | 0 | 82 | 82 |
| ELOVL5 | NM_021814 | 5 | 5 | 0 | 5 | 5 |
| PIP4K2A | NM_005028 | 16 | 16 | 0 | 16 | 16 |
| SPEN | NM_015001 | 10 | 10 | 0 | 10 | 10 |
| PAWR | NM_002583 | 16 | 16 | 0 | 16 | 16 |
| YTHDF3 | NM_152758 | 43 | 43 | 0 | 43 | 43 |
| EIF2C1 | NM_012199 | 3 | 3 | 0 | 3 | 3 |
| HIC2 | NM_015094 | 46 | 46 | 0 | 46 | 46 |

| | | | | | | | |
|---|---|---|---|---|---|---|---|
| | PPTC7 | NM_139283 | 3 | 3 | 0 | 3 | 0 |
| | PTP4A1 | NM_003463 | 12 | 12 | 0 | 12 | 0 |
| | CHMP2B | NM_014043 | 13 | 13 | 0 | 13 | 0 |
| | EDC3 | NM_001142443 | 7 | 7 | 0 | 7 | 0 |
| | EML4 | NM_001145076 | 10 | 10 | 0 | 10 | 0 |
| | EED | NM_003797 | 6 | 6 | 0 | 6 | 0 |
| | SERPINE1 | NM_000602 | 1 | 1 | 0 | 1 | 0 |
| | GOLGA4 | NM_001172713 | 49 | 49 | 0 | 49 | 0 |
| | ARID3A | NM_005224 | 3 | 3 | 0 | 0 | 3 |
| | RAPH1 | NM_213589 | 5 | 5 | 0 | 0 | 5 |
| | PTGFRN | NM_020440 | 23 | 23 | 0 | 0 | 23 |
| | AZIN1 | NM_148174 | 26 | 26 | 0 | 0 | 0 |
| | SATB1 | NM_002971 | 3 | 3 | 0 | 0 | 0 |
| | PDCD10 | NM_145859 | 7 | 7 | 0 | 0 | 0 |
| | BACH1 | NM_206866 | 16 | 16 | 0 | 0 | 0 |
| | TBPL1 | NM_004865 | 2 | 2 | 0 | 0 | 0 |
| | BCL11A | NM_018014 | 3 | 3 | 0 | 0 | 0 |
| | IDH1 | NM_005896 | 10 | 10 | 0 | 0 | 0 |
| | SLC6A6 | NM_003043 | 7 | 7 | 0 | 0 | 0 |
| | E2F7 | NM_203394 | 46 | 46 | 0 | 0 | 0 |
| | E2F7 | NM_203394 | 64 | 64 | 0 | 0 | 0 |
| | CCNT2 | NM_058241 | 14 | 14 | 0 | 0 | 0 |
| | DLG5 | NM_004747 | 2 | 2 | 0 | 0 | 0 |
| | NRBF2 | NM_030759 | 1 | 1 | 0 | 0 | 0 |
| | PTGFRN | NM_020440 | 23 | 23 | 0 | 0 | 0 |
| | SPEN | NM_015001 | 34 | 34 | 0 | 0 | 0 |
| | PHF13 | NM_153812 | 18 | 18 | 0 | 0 | 0 |
| | GNA13 | NM_006572 | 40 | 40 | 0 | 0 | 0 |
| | FAM160B1 | NM_020940 | 3 | 0 | 3 | 0 | 3 |
| | LIFR | NM_001127671 | 148 | 0 | 0 | 148 | 148 |
| | QKI | NM_206853 | 218 | 0 | 0 | 218 | 218 |
| | SIX4 | NM_017420 | 31 | 0 | 0 | 31 | 31 |
| | TFDP1 | NR_026580 | 7 | 0 | 0 | 7 | 7 |
| | C13orf37 | NM_001071775 | 61 | 0 | 0 | 61 | 61 |
| | MLL3 | NM_170606 | 2 | 0 | 0 | 2 | 2 |
| | JAK1 | NM_002227 | 8 | 0 | 0 | 8 | 8 |
| | TNRC6B | NM_001162501 | 3 | 0 | 0 | 3 | 3 |
| | TNRC6B | NM_001162501 | 8 | 0 | 0 | 8 | 8 |
| | CBX3 | NM_016587 | 11 | 0 | 0 | 11 | 11 |
| | SP4 | NM_003112 | 13 | 0 | 0 | 13 | 13 |
| | NFYB | NM_006166 | 2 | 0 | 0 | 2 | 2 |
| | PIP4K2B | NM_003559 | 4 | 0 | 0 | 4 | 4 |
| | ZNF238 | NM_205768 | 15 | 0 | 0 | 15 | 15 |
| | ZSCAN29 | NM_152455 | 2 | 0 | 0 | 2 | 2 |
| | ZNF507 | NM_014910 | 20 | 0 | 0 | 20 | 20 |
| | SETD5 | NM_001080517 | 7 | 0 | 0 | 7 | 8 |
| | PLAGL2 | NM_002657 | 6 | 0 | 0 | 6 | 6 |
| | KLHL28 | NM_017658 | 10 | 0 | 0 | 10 | 10 |
| | PLEKHO2 | NM_025201 | 2 | 0 | 0 | 2 | 2 |
| | ZBTB41 | NM_194314 | 9 | 0 | 0 | 9 | 9 |
| | FRS2 | NM_006654 | 8 | 0 | 0 | 8 | 8 |

| | | | | | | |
|---|---|---|---|---|---|---|
| C10orf26 | NM_001083913 | 2 | 0 | 0 | 2 | 2 |
| FAM91A1 | NM_144963 | 1 | 0 | 0 | 1 | 1 |
| KIAA0247 | NM_014734 | 3 | 0 | 0 | 3 | 3 |
| CALD1 | NM_033138 | 2 | 0 | 0 | 2 | 2 |
| RAP2B | NM_002886 | 14 | 0 | 0 | 14 | 14 |
| TXNDC5 | NM_001145549 | 17 | 0 | 0 | 17 | 17 |
| PHACTR2 | NM_001100166 | 10 | 0 | 0 | 10 | 10 |
| RFX7 | NM_022841 | 1 | 0 | 0 | 1 | 1 |
| TNRC6A | NM_014494 | 2 | 0 | 0 | 2 | 2 |
| ODZ3 | NM_001080477 | 9 | 0 | 0 | 9 | 9 |
| NEFL | NM_006158 | 1 | 0 | 0 | 1 | 1 |
| LIN28B | NM_001004317 | 10 | 0 | 0 | 10 | 10 |
| GLCCI1 | NM_138426 | 12 | 0 | 0 | 12 | 12 |
| PRKRIR | NM_004705 | 2 | 0 | 0 | 2 | 2 |
| FRZB | NM_001463 | 8 | 0 | 0 | 8 | 8 |
| GNPDA1 | NM_005471 | 9 | 0 | 0 | 9 | 9 |
| CD2AP | NM_012120 | 11 | 0 | 0 | 11 | 11 |
| CEP350 | NM_014810 | 3 | 0 | 0 | 3 | 3 |
| ZBTB39 | NM_014830 | 24 | 0 | 0 | 24 | 24 |
| FAM104A | NM_032837 | 21 | 0 | 0 | 21 | 21 |
| MAF | NM_001031804 | 4 | 0 | 0 | 4 | 4 |
| CHD1 | NM_001270 | 26 | 0 | 0 | 26 | 26 |
| FAM81A | NM_152450 | 3 | 0 | 0 | 3 | 3 |
| FBXO45 | NM_001105573 | 7 | 0 | 0 | 7 | 0 |
| UBN2 | NM_173569 | 19 | 0 | 0 | 19 | 0 |
| WDR82 | NM_025222 | 7 | 0 | 0 | 7 | 0 |
| PRPF40A | NM_017892 | 10 | 0 | 0 | 10 | 0 |
| SH2B3 | NM_005475 | 11 | 0 | 0 | 11 | 0 |
| KIAA1211 | NM_020722 | 3 | 0 | 0 | 3 | 0 |
| RAB23 | NM_183227 | 6 | 0 | 0 | 6 | 0 |
| UBE3C | NM_014671 | 3 | 0 | 0 | 3 | 0 |
| KPNA6 | NM_012316 | 3 | 0 | 0 | 3 | 0 |
| ZBTB39 | NM_014830 | 5 | 0 | 0 | 5 | 0 |
| C13orf37 | NM_001071775 | 4 | 0 | 0 | 0 | 4 |
| S100PBP | NM_022753 | 7 | 0 | 0 | 0 | 7 |
| SNX10 | NM_013322 | 1 | 0 | 0 | 0 | 1 |
| BDP1 | NM_018429 | 7 | 0 | 0 | 0 | 7 |
| C1orf135 | NM_024037 | 1 | 0 | 0 | 0 | 1 |
| SLC16A14 | NM_152527 | 2 | 0 | 0 | 0 | 2 |
| KLF9 | NM_001206 | 0 | 1 | 0 | 1 | 10 |
| YPEL5 | NM_016061 | 0 | 4 | 0 | 4 | 4 |
| TMED2 | NM_006815 | 0 | 7 | 0 | 7 | 7 |
| KRAS | NM_033360 | 0 | 9 | 0 | 9 | 9 |
| MIER3 | NM_152622 | 0 | 17 | 0 | 17 | 17 |
| RAB11A | NM_004663 | 0 | 5 | 0 | 5 | 5 |
| SATB2 | NM_015265 | 0 | 2 | 0 | 2 | 8 |
| GLCE | NM_015554 | 0 | 6 | 0 | 6 | 0 |
| NAA25 | NM_024953 | 0 | 10 | 0 | 0 | 10 |
| LEPROTL1 | NM_001128208 | 0 | 0 | 7 | 0 | 7 |
| E2F3 | NM_001949 | 0 | 0 | 4 | 0 | 4 |
| TXNDC12 | NM_015913 | 0 | 0 | 11 | 0 | 11 |

| | | | | | | | |
|---|---|---|---|---|---|---|---|
| | CAPRIN2 | NM_001002259 | 0 | 0 | 15 | 0 | 15 |
| | SLC39A10 | NM_001127257 | 0 | 0 | 0 | 109 | 109 |
| | SEC24A | NM_021982 | 0 | 0 | 0 | 7 | 7 |
| | STOX2 | NM_020225 | 0 | 0 | 0 | 8 | 8 |
| | CCNY | NM_145012 | 0 | 0 | 0 | 4 | 4 |
| | NRBP1 | NM_013392 | 0 | 0 | 0 | 1 | 1 |
| | HNRNPC | NM_031314 | 0 | 0 | 0 | 3 | 3 |
| | TMEM33 | NM_018126 | 0 | 0 | 0 | 3 | 3 |
| | TGDS | NM_014305 | 0 | 0 | 0 | 5 | 5 |
| | ZFX | NM_003410 | 0 | 0 | 0 | 3 | 3 |
| | GOLGA8B | NM_001023567 | 0 | 0 | 0 | 2 | 2 |
| | ZNF711 | NM_021998 | 0 | 0 | 0 | 3 | 3 |
| | IGF1R | NM_000875 | 0 | 0 | 0 | 4 | 4 |
| | CLTC | NM_004859 | 0 | 0 | 0 | 5 | 5 |
| 99a-5p | MBNL1 | NM_207297 | 19 | 19 | 0 | 19 | 0 |
| | SMARCA5 | NM_003601 | 19 | 19 | 0 | 19 | 19 |
| | FZD8 | NM_031866 | 6 | 6 | 0 | 6 | 291 |
| | VLDLR | NM_003383 | 0 | 14 | 14 | 0 | 0 |
| | TRIB2 | NR_027303 | 9 | 9 | 0 | 9 | 9 |
| | EIF2C2 | NM_012154 | 22 | 22 | 0 | 22 | 22 |
| | KBTBD8 | NM_032505 | 21 | 21 | 0 | 21 | 21 |
| | ZZEF1 | NM_015113 | 2 | 2 | 0 | 2 | 2 |
| | BAZ2A | NM_013449 | 3 | 3 | 0 | 3 | 3 |
| | HS3ST3B1 | NM_006041 | 6 | 6 | 0 | 6 | 6 |
| 100-5p | MBNL1 | NM_207297 | 19 | 19 | 0 | 19 | 0 |
| | SMARCA5 | NM_003601 | 19 | 19 | 0 | 19 | 19 |
| | FZD8 | NM_031866 | 6 | 6 | 0 | 6 | 291 |
| | VLDLR | NM_003383 | 0 | 14 | 14 | 0 | 0 |
| | TRIB2 | NR_027303 | 9 | 9 | 0 | 9 | 9 |
| | EIF2C2 | NM_012154 | 22 | 22 | 0 | 22 | 22 |
| | KBTBD8 | NM_032505 | 21 | 21 | 0 | 21 | 21 |
| | ZZEF1 | NM_015113 | 2 | 2 | 0 | 2 | 2 |
| | BAZ2A | NM_013449 | 3 | 3 | 0 | 3 | 3 |
| | HS3ST3B1 | NM_006041 | 6 | 6 | 0 | 6 | 6 |
| 122-5p | SOX4 | NM_003107 | 0 | 0 | 31 | 0 | 31 |
| | ZCCHC3 | NM_033089 | 0 | 0 | 5 | 0 | 5 |
| | METTL9 | NM_001077180 | 0 | 0 | 11 | 11 | 11 |
| | GIT1 | NM_014030 | 2 | 2 | 0 | 0 | 2 |
| | FUBP3 | NM_003934 | 0 | 0 | 4 | 0 | 4 |
| | CALM3 | NM_005184 | 1 | 1 | 0 | 1 | 1 |
| | ATP1B1 | NM_001001787 | 0 | 0 | 7 | 0 | 7 |
| | STK24 | NM_003576 | 0 | 8 | 0 | 0 | 47 |
| | COLEC12 | NM_130386 | 0 | 0 | 8 | 0 | 8 |
| | CPEB4 | NM_030627 | 0 | 0 | 7 | 0 | 7 |
| | KIAA0174 | NM_014761 | 0 | 0 | 0 | 2 | 2 |
| | TNRC6A | NM_014494 | 0 | 34 | 0 | 34 | 34 |
| | CCAR1 | NM_018237 | 0 | 0 | 0 | 11 | 11 |
| | LAMC1 | NM_002293 | 2 | 2 | 0 | 2 | 2 |
| | ABCF2 | NM_005692 | 0 | 0 | 0 | 62 | 62 |
| | PIP4K2A | NM_005028 | 15 | 0 | 0 | 15 | 0 |
| | RBM47 | NM_019027 | 2 | 2 | 0 | 0 | 0 |

| | | | | | | | |
|---|---|---|---|---|---|---|---|
| | LMNB2 | NM_032737 | 48 | 0 | 0 | 48 | 48 |
| | RNF145 | NM_144726 | 0 | 0 | 291 | 0 | 291 |
| 192-5p | NCOA3 | NM_001174087 | 9 | 0 | 0 | 9 | 9 |
| | DICER1 | NM_177438 | 6 | 0 | 0 | 6 | 6 |
| | NKRF | NM_017544 | 0 | 8 | 9 | 0 | 0 |
| | SH3RF3 | NM_001099289 | 2 | 0 | 0 | 2 | 0 |
| | WNK1 | NM_018979 | 5 | 0 | 0 | 5 | 0 |
| | RICTOR | NM_152756 | 4 | 0 | 0 | 4 | 4 |
| | PABPC4 | NM_003819 | 12 | 0 | 0 | 12 | 0 |
| 194-5p | KPNA1 | NR_026698 | 42 | 0 | 0 | 42 | 42 |
| | PAN3 | NM_175854 | 0 | 0 | 3 | 0 | 3 |
| | EHBP1 | NM_015252 | 0 | 2 | 0 | 2 | 2 |
| | PRKAR1A | NM_002734 | 3 | 0 | 3 | 3 | 3 |
| | MIB1 | NM_020774 | 4 | 0 | 0 | 4 | 4 |
| | CAV1 | NM_001172897 | 0 | 0 | 0 | 6 | 6 |
| | EPC2 | NM_015630 | 11 | 11 | 0 | 11 | 11 |
| | ZBTB10 | NM_023929 | 0 | 0 | 0 | 5 | 5 |
| | PTBP1 | NM_031990 | 0 | 0 | 0 | 17 | 17 |
| | FAM122B | NM_001170757 | 0 | 0 | 12 | 0 | 12 |
| | ACVR1 | NM_001111067 | 2 | 0 | 0 | 2 | 2 |
| | KCMF1 | NM_020122 | 2 | 2 | 0 | 0 | 0 |
| | PAIP2 | NM_016480 | 0 | 7 | 0 | 7 | 7 |
| | BTF3L4 | NM_001136497 | 0 | 0 | 0 | 62 | 62 |
| | NAA50 | NM_025146 | 4 | 4 | 0 | 4 | 4 |
| | CALU | NM_001130674 | 0 | 0 | 0 | 13 | 13 |
| | HOXB3 | NM_002146 | 0 | 0 | 0 | 1 | 1 |
| | CSNK1D | NM_139062 | 0 | 9 | 0 | 9 | 9 |
| | SETD5 | NM_001080517 | 2 | 0 | 0 | 2 | 2 |
| | PAXIP1 | NM_007349 | 0 | 0 | 0 | 19 | 19 |
| | SPCS2 | NM_014752 | 7 | 7 | 0 | 7 | 7 |
| | PURB | NM_033224 | 0 | 0 | 0 | 3 | 3 |
| | RASL10B | NM_033315 | 0 | 0 | 0 | 1 | 1 |
| | PSME3 | NM_176863 | 10 | 0 | 0 | 0 | 10 |
| | SPPL3 | NM_139015 | 0 | 0 | 0 | 4 | 4 |
| | GIGYF1 | NM_022574 | 286 | 286 | 0 | 0 | 0 |
| | CAPZA1 | NM_006135 | 0 | 0 | 0 | 37 | 37 |
| | TMED5 | NR_030761 | 0 | 0 | 0 | 4 | 4 |
| | RBM39 | NM_184234 | 0 | 0 | 0 | 3 | 3 |
| | TSPAN3 | NM_198902 | 0 | 0 | 0 | 5 | 5 |
| | SLK | NM_014720 | 0 | 0 | 0 | 3 | 3 |
| | BTBD7 | NM_001002860 | 2 | 0 | 0 | 2 | 2 |
| | TNRC6A | NM_014494 | 0 | 0 | 0 | 7 | 7 |
| | SLC35F1 | NM_001029858 | 0 | 0 | 0 | 2 | 2 |
| | PFN2 | NM_002628 | 29 | 29 | 0 | 0 | 0 |
| | MAPK1 | NM_002745 | 10 | 0 | 0 | 10 | 0 |
| | BMI1 | NM_005180 | 0 | 0 | 11 | 11 | 11 |
| | BNIP2 | NM_004330 | 19 | 0 | 0 | 0 | 19 |
| | DCAF7 | NM_005828 | 0 | 0 | 0 | 17 | 17 |
| | DEPDC1B | NM_018369 | 2 | 2 | 0 | 2 | 0 |
| | REV3L | NM_002912 | 39 | 39 | 0 | 0 | 0 |
| | MYST4 | NM_012330 | 0 | 0 | 0 | 12 | 12 |

| | | | | | | | |
|---|---|---|---|---|---|---|---|
| | RBX1 | NM_014248 | 0 | 0 | 0 | 38 | 38 |
| | TJAP1 | NM_001146017 | 5 | 5 | 0 | 5 | 5 |
| | SSH2 | NM_033389 | 7 | 7 | 0 | 0 | 7 |
| | RAB5A | NM_004162 | 0 | 0 | 0 | 9 | 9 |
| | AASDHPPT | NM_015423 | 0 | 0 | 0 | 10 | 10 |
| 605 | TMEM30A | NM_018247 | 0 | 0 | 0 | 10 | 10 |
| | ATXN1 | NM_000332 | 0 | 0 | 0 | 4 | 4 |
| | PPP4R1 | NM_001042388 | 0 | 0 | 0 | 114 | 114 |
| | TNRC6B | NM_001162501 | 0 | 0 | 0 | 3 | 3 |
| | C5orf13 | NM_001142476 | 0 | 0 | 0 | 4 | 4 |
| | SFRS7 | NM_001031684 | 0 | 0 | 0 | 28 | 28 |
| | QSOX2 | NM_181701 | 0 | 0 | 0 | 6 | 6 |
| | PTGES3 | NM_006601 | 0 | 0 | 0 | 3 | 4 |
| | CADM1 | NM_001098517 | 0 | 0 | 0 | 8 | 8 |
| | ADIPOR2 | NM_024551 | 0 | 0 | 0 | 17 | 17 |
| | CSNK1A1 | NM_001025105 | 0 | 0 | 0 | 27 | 27 |
| | SMC1A | NM_006306 | 0 | 0 | 0 | 5 | 5 |
| | H3F3B | NM_005324 | 0 | 0 | 0 | 10 | 10 |
| | E2F7 | NM_203394 | 0 | 0 | 0 | 64 | 64 |
| | RUNX1T1 | NM_175636 | 0 | 0 | 0 | 4 | 4 |
| | ODC1 | NM_002539 | 0 | 0 | 0 | 9 | 9 |
| | NLK | NM_016231 | 0 | 0 | 0 | 14 | 14 |
| | ZCCHC14 | NM_015144 | 0 | 0 | 0 | 7 | 7 |
| | LIN28B | NM_001004317 | 0 | 0 | 0 | 87 | 87 |
| | ZDHHC21 | NM_178566 | 0 | 0 | 0 | 8 | 8 |
| | PDIK1L | NR_026685 | 0 | 0 | 0 | 3 | 4 |
| | SCD | NM_005063 | 0 | 0 | 0 | 1348 | 1348 |
| | SOX9 | NM_000346 | 0 | 0 | 0 | 7 | 7 |
| | SEC24D | NM_014822 | 0 | 0 | 0 | 4 | 6 |
| | BRWD1 | NM_033656 | 0 | 0 | 0 | 8 | 8 |
| | WDR26 | NM_001115113 | 0 | 0 | 0 | 91 | 91 |
| | ZFHX3 | NM_006885 | 0 | 0 | 0 | 3 | 3 |
| | GCLM | NM_002061 | 0 | 0 | 0 | 8 | 8 |
| | JHDM1D | NM_030647 | 0 | 0 | 0 | 4 | 4 |
| 636 | MSRB3 | NM_198080 | 0 | 0 | 0 | 5 | 5 |
| | PRKACB | NM_002731 | 0 | 0 | 0 | 22 | 22 |
| | LRP12 | NM_001135703 | 0 | 0 | 0 | 19 | 19 |
| | FAM98A | NM_015475 | 0 | 0 | 0 | 2 | 2 |
| | ARID4B | NM_016374 | 0 | 0 | 0 | 2 | 2 |
| | KDM2A | NR_027473 | 0 | 0 | 0 | 3 | 3 |
| | CRIM1 | NM_016441 | 0 | 0 | 0 | 5 | 7 |
| | SUZ12 | NM_015355 | 0 | 0 | 0 | 3 | 3 |
| | SSR1 | NM_003144 | 0 | 0 | 0 | 16 | 16 |
| | FBXO30 | NM_032145 | 0 | 0 | 0 | 30 | 30 |
| | C10orf12 | NM_015652 | 0 | 0 | 0 | 4 | 4 |
| | CAPZA1 | NM_006135 | 0 | 0 | 0 | 2 | 2 |
| | ETF1 | NM_004730 | 0 | 0 | 0 | 3 | 3 |
| | TOB1 | NM_005749 | 0 | 0 | 0 | 2 | 2 |
| | YTHDF3 | NM_152758 | 0 | 0 | 0 | 21 | 21 |
| 885-5p | MEX3B | NM_032246 | 0 | 0 | 0 | 1 | 5 |
| | SIX4 | NM_017420 | 0 | 0 | 0 | 40 | 40 |

| | | | | | | |
|---|---|---|---|---|---|---|
| TUBB2B | NM_178012 | 0 | 0 | 0 | 7 | 7 |
| CREBZF | NR_028027 | 0 | 0 | 0 | 7 | 7 |
| MCL1 | NM_021960 | 0 | 0 | 0 | 5 | 5 |
| ATP1B1 | NM_001001787 | 0 | 0 | 0 | 3 | 3 |
| YWHAE | NR_024058 | 0 | 0 | 0 | 1 | 1 |
| DAZAP2 | NM_001136264 | 0 | 0 | 0 | 25 | 25 |
| EIF4G2 | NM_001042559 | 0 | 0 | 0 | 133 | 133 |
| SYPL1 | NM_182715 | 0 | 0 | 0 | 340 | 340 |
| MIER3 | NM_152622 | 0 | 0 | 0 | 73 | 73 |
| ING3 | NM_019071 | 0 | 0 | 0 | 2 | 2 |
| IGF1R | NM_000875 | 0 | 0 | 0 | 34 | 34 |
| YTHDF3 | NM_152758 | 0 | 0 | 0 | 4 | 4 |
| KDM6A | NM_021140 | 0 | 0 | 0 | 8 | 8 |
| MRPS25 | NM_022497 | 0 | 0 | 0 | 29 | 29 |
| ZNF281 | NM_012482 | 0 | 0 | 0 | 2 | 3 |
| PAPD4 | NM_173797 | 0 | 0 | 0 | 17 | 17 |
| PGRMC2 | NM_006320 | 0 | 0 | 0 | 17 | 17 |
